# Biotechnological Applications of Microbial (Per)chlorate Reduction

**DOI:** 10.3390/microorganisms5040076

**Published:** 2017-11-24

**Authors:** Ouwei Wang, John D. Coates

**Affiliations:** 1 Department of Plant and Microbial Biology, University of California, Berkeley, CA 94720, USA; wangouwei@berkeley.edu; 2 Energy Biosciences Institute, University of California, Berkeley, CA 94704, USA; 3 Environmental Genomics and Systems Biology Division, Lawrence Berkeley National Laboratory, Berkeley, CA 94720, USA

**Keywords:** perchlorate, chlorate, microbial perchlorate reduction, perchlorate bioremediation, bio-souring control, bioreactor hygiene controls, aeration enhancement

## Abstract

While the microbial degradation of a chloroxyanion-based herbicide was first observed nearly ninety years ago, only recently have researchers elucidated the underlying mechanisms of perchlorate and chlorate [collectively, (per)chlorate] respiration. Although the obvious application of these metabolisms lies in the bioremediation and attenuation of (per)chlorate in contaminated environments, a diversity of alternative and innovative biotechnological applications has been proposed based on the unique metabolic abilities of dissimilatory (per)chlorate-reducing bacteria (DPRB). This is fueled in part by the unique ability of these organisms to generate molecular oxygen as a transient intermediate of the central pathway of (per)chlorate respiration. This ability, along with other novel aspects of the metabolism, have resulted in a wide and disparate range of potential biotechnological applications being proposed, including enzymatic perchlorate detection; gas gangrene therapy; enhanced xenobiotic bioremediation; oil reservoir bio-souring control; chemostat hygiene control; aeration enhancement in industrial bioreactors; and, biogenic oxygen production for planetary exploration. While previous reviews focus on the fundamental science of microbial (per)chlorate reduction (for example see Youngblut et al., 2016), here, we provide an overview of the emerging biotechnological applications of (per)chlorate respiration and the underlying organisms and enzymes to environmental and biotechnological industries.

## 1. Introduction

The oxyanions of chlorine, perchlorate (ClO_4_^−^), and chlorate (ClO_3_^−^), are highly soluble, strong oxidants that are deposited in the environment through both anthropogenic and natural processes [1,2,3,4,5,6,7,8]. Perchlorate is a common commodity chemical with a diverse range of industrial uses, ranging from pyrotechnics to lubricating oils [4], but it is predominantly used as an energetics booster or oxidant in solid rocket fuels by the munitions industry [4,9,10,11]. Although being a powerful oxidant, under most environmental conditions perchlorate is highly stable on account of the high energy of activation that is associated with its reduction [1,9]. Perchlorate is toxic to humans as it inhibits the uptake of iodine by the thyroid gland [12], disrupting the production of thyroid hormones, potentially leading to hypothyroidism [13,14]. For humans, oral ingestion is the primary source of perchlorate exposure as contamination is widespread in soil, fertilizers, and groundwater, allowing it to readily move into our food chain [15]. 

In contrast, chlorate is far more chemically reactive than perchlorate [16]. It is currently used to produce chlorine dioxide in bleaching and disinfection processes [17] and as a precursor for the manufacture of perchlorate. Historically, it was widely used as the active component of herbicides during the early twentieth century [18,19]. Chlorate is also toxic to humans causing oxidative damage to red blood cells, resulting in methemoglobin formation [20,21]. In both plant and microbial cells, chlorate can be a substrate for respiratory or assimilatory nitrate reductases, which reduce it to chlorite (ClO_2_^−^) [22], a compound with demonstrated broad-spectrum biocidal activity against Gram-negative and Gram-positive bacteria [23,24,25,26,27,28], bacteriophages [29], fungi [23], and algae [25]. Because of this, chlorite is often used to treat freshwater copepod parasites [30], and as an active ingredient in topical wound treatments. 

**Figure 1:**
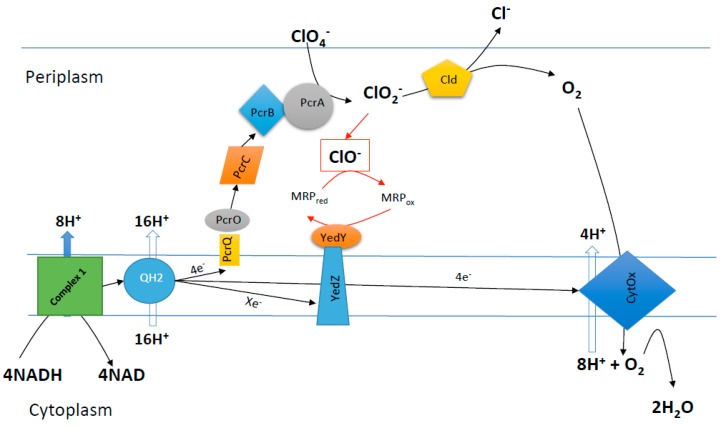
A schematic of the current model for the biochemical pathway of perchlorate reduction. As part of this pathway, hypochlorite (ClO^−^) is inadvertently produced at the chlorite dismutase. Perchlorate reducing bacteria have evolved a detoxification process based on a methionine rich peptide that chemically reacts with ClO^−^ to produce methionine sulfoxide, which is subsequently re-reduced by the methionine sulfoxide reductase (YedY) using reducing equivalents from the quinone oxidoreductase (YedZ). QH—quinone pool; Pcr—Perchlorate reductase; Cld—Chlorite dismutase; CytOx—Cytochrome oxidase; and, MRP—methionine rich peptide. For the biochemical pathway of chlorate reduction, PcrABC is replaced by chlorate reductase ClrABC, however, the process by which ClrABC receives electrons from the quinone pool is currently not well understood.

In the 1920’s, bacteria were discovered that used chlorate, (and presumably perchlorate, although this is not a prerequisite [18]) as a terminal electron acceptor for anaerobic energy production [31]. Recent studies have identified the molecular mechanisms underlying this unique biochemistry (Figure 1) [9,18,19]. Dissimilatory (per)chlorate reducing bacteria (DPRB) use a highly conserved perchlorate reductase, PcrABC, to reduce perchlorate to chlorate and subsequently chlorite. The chlorite is rapidly removed by another highly conserved enzyme, chlorite dismutase (Cld), to produce molecular oxygen (O_2_) and innocuous chloride (Cl^−^) [9,18,19,32]. The oxygen that is produced is then respired by the same organism, generally through the use of a high-affinity cytochrome *cbb3*-oxidase [18,19]. Specialized dissimilatory chlorate-reducing bacteria (DCRB) respire only chlorate and not perchlorate using an analogous pathway involving chlorite and Cld, but the chlorate is initially reduced via a specialized chlorate reductase (ClrABC), a structurally and evolutionarily distinct enzyme from the PcrABC that can reduce chlorate but not perchlorate [33,34,35,36,37]. Owing to the unique ability to degrade (per)chlorate and generate molecular oxygen under anaerobic conditions, DPRB, perchlorate reductase, chlorate reductase, and chlorite dismutase provide innovative solutions to outstanding problems in current industrial processes. In this review, we provide an overview of the potential applications of microbial (per)chlorate reduction, and the underlying genes and enzymes. The taxonomy, evolution, biochemistry, and physiology of microbial (per)chlorate reduction are beyond the scope of this review and have been described in detail elsewhere [18,19].

## 2. (Per)chlorate Bioremediation 

The primary application of microbial (per)chlorate reduction is the bio-attenuation of perchlorate-contaminated water sources [38]. Bioremediation of perchlorate can be much more effective than other remediation strategies, as perchlorate is completely and irreversibly reduced to innocuous chloride [38]. For example, although ion exchange based technology is a widely used and effective method for perchlorate removal, it does not uniquely bind to perchlorate, rather the ion exchange resins also bind other common co-contaminating anions such as nitrate which are often present in concentrations several orders of magnitude higher than perchlorate. As such, much of the ion exchange capacity is lost in binding these analogous compounds [38]. Furthermore, ion exchange technologies concentrate perchlorate and other ions into a recalcitrant brine, which often poses disposal issues in its own right [19,38,39,40]. In contrast, perchlorate metabolism by DPRB completely converts perchlorate into innocuous chloride [38]. This can be used as an independent treatment strategy or can be applied in combination with ion exchange methods using halophilic DPRB [41,42,43,44] or salt-tolerant communities [39,45,46,47] to treat perchlorate-laden waste brines that are extracted from ion exchange resins that would otherwise pose significant health and disposal issues.

Perchlorate bioremediation can be achieved in-situ or ex-situ using various types of bioreactors. While laboratory-scale autotrophic reactors using hydrogen [48,49,50,51], reduced iron [52], or sulfur compounds [53] as the electron donor have been developed, most industrial-scale fixed or fluidized-bed reactors are operated as heterotrophic reactors using either simple alcohols or acetate as the electron donor [19,54]. Among the different types, fluidized-bed heterotrophic (with ethanol as the electron donor) bioreactors have emerged as the most successful for perchlorate treatment with capacities up to 34 × 10^6^ L·day^−1^, reducing perchlorate concentrations to below 6 ppb (6 μg·L^−1^), the maximum concentration limit that is allowed in the state of California [19,55].

**Figure 2:**
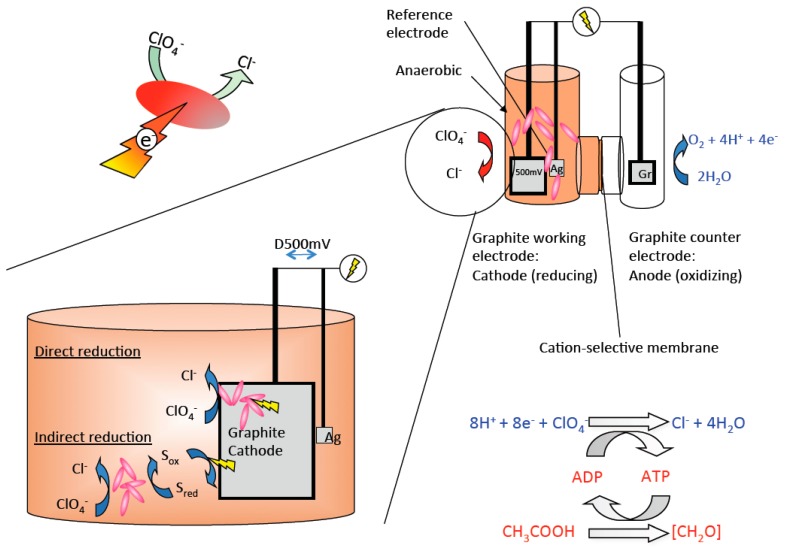
A schematic diagram of a packed-bed bioelectrical reactor for perchlorate bioremediation. DPRB use electrons provided through a negatively charged cathode as the direct or indirect (mediated by an electron shuttle) electron donor to reduce perchlorate to innocuous chloride. S ox—oxidized electron shuttle; S red—reduced electron shuttle.

Conventional bioreactors always depend on the continuous addition of a chemical electron donor and are subject to bio-fouling issues due to microbial growth [54,56,57,58]. Furthermore, disposal of the excess biomass generated represents a significant operational cost of the treatment process, while any residual labile electron donor in the reactor effluent can stimulate microbial growth in water distribution systems and contribute to the formation of toxic trihalomethanes during disinfection by chlorination [59]. These issues led to the proposal of a diversity of alternative advanced reactor designs. An innovative and unorthodox bioreactor concept was developed in 2007 based on the electrochemical stimulation of microbial perchlorate reduction [56,60,61] (Figure 2). In these packed-bed bioelectrical reactors, DPRB use electrons that are provided through a negatively charged cathode set at a redox potential of 500 mV as an electron donor. Preliminary studies suggested that electrons were either directly consumed off the electrode surface or indirectly through the use of anthraquinone-2,6-disulfonate as a recyclable electron shuttle [56,62]. However, subsequently, it was demonstrated that these reactors did not function mechanistically as initially perceived [62]. Remarkably, it was shown that living DPRB cells were not required for perchlorate reduction in the bioelectrical reactors. Rather, catalytic amounts of oxygen contaminating the bioelectrical packed-bed reactors reacted with electrochemically reduced anthraquinone-2,6-disulfonate to form superoxide and hydrogen peroxide, leading to DPRB cell lysis and active enzyme release [62]. The observed perchlorate reduction and removal was primarily mediated through interconnected enzymatic electrocatalysis mechanisms, in which the cathode-reduced anthraquinone-2,6-disulfonate functioned as a direct electron donor for the perchlorate reductase enzyme PcrAB that was released from the dying lysing microbial cells [56,62]. While the underlying mechanism of perchlorate attenuation was not dependent on living cells, the technology still shows great promise and could be improved by the use of nanocarbon conductive interlinks between the electrode surface and the cell or by the identification of DPRB that can use electrons directly off the electrode surface similar to the mechanism used by a diversity of iron reducing species [63,64]. Alternatively, the redox potential on the cathode could be increased to allow for electrochemical H_2_ generation, which could support an autotrophic DPRB population. Although further work is needed to develop the technology to commercial application, the advantages are substantial in being able to control the rate of metabolism and perchlorate treatment independently from biomass growth (Figure 2).

## 3. Enzymatic Bioassay for the Detection of Perchlorate 

**Figure 3:**
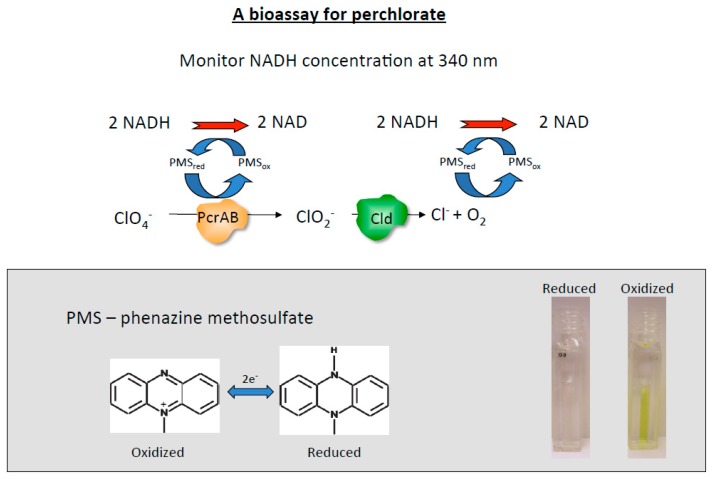
Schematic for the high throughput bioassay (with a perchlorate detection range of 2–17,000 ppb) using perchlorate reductase (PcrAB), chlorite dismutase (Cld), nicotine adenine dinucleotide (NADH), and the electron shuttle phenazine methosulfate (PMS).

Ion chromatography and mass spectrometry are commonly used for the detection and quantification of perchlorate in environmental samples [65,66,67]. Although sensitive, these methods are slow, costly, and require specialized personnel and laboratories. Therefore, an enzymatic bioassay that offers a cost-effective alternative with the potential for high throughput application was developed using a mixture of purified perchlorate reductase (PcrAB), nicotine adenine dinucleotide (NADH), and the electron shuttle phenazine methosulfate (PMS) [68,69]. In this bioassay, PMS mediates the transfer of electrons from NADH to PcrAB, which subsequently reduces perchlorate to chlorite (Figure 3). The enzymatically produced chlorite is subsequently dismutated by Cld to produce O_2_, which chemically reacts with the PMS resulting in further NADH oxidation. This additional PMS cycle provides an effective twofold signal amplification (Figure 3). The perchlorate concentration is indirectly measured by the oxidation of NADH, which is monitored as absorbance changes at 340 nm [68,69]. A perchlorate detection range of 2–17,000 ppb (μg·L^−1^) was achieved when the bioassay was coupled with a solid-phase extraction step to purify and concentrate the perchlorate [68,69]. This step, based on styrene divinyl benzene syringe columns pretreated with decyltrimethylammonium bromide (DTAB), allowed for the selective extraction and concentration of perchlorate from solutions with numerous co-occurring ions including nitrate, phosphate, sulfate, iron, and chloride [68,69]. Although this bioassay is sensitive to air due to the slow oxygen-induced inactivation of perchlorate reductase, this issue was avoided by covering all of the reactions with mineral oil [69]. Alternatively, an oxygen insensitive enzyme, such as suitable nitrate reductase that is capable of reducing perchlorate, could conceptually be used to replace the PcrAB, although this might significantly impact the assay lower limit of detection. Youngblut et al. [32] recently demonstrated that the PcrAB is a specialized member of the DMSO reductase superfamily, with a relatively high perchlorate affinity (*K*_M_ = 6 μM), unlike the closely related nitrate reductase, which has a much lower affinity for perchlorate (*K*_M_ = 1.1 mM). 

## 4. Bioeletrochemical O_2_ Production from Perchlorate

Perchlorate has been detected and inferred on Mars at concentrations between 0.5% and 1% [70,71]. Similarly, perchlorate has also been detected in lunar and meteorite samples [72], suggesting its prevalence across the solar system and possibly beyond. Due to unrestricted UV radiation and the lack of biotic perchlorate diagenesis processes, the quantity of perchlorate in the surface regolith of Mars is three to four orders of magnitude higher when compared with soils on Earth, and offers an attractive resource for human exploration and survival. Traditionally, perchlorate is used as an energetic booster or oxidant in solid rocket fuel. However, perchlorate on Mars could also serve as a source of O_2_ for human consumption [73]. For example, humans respire 550–650 L of oxygen per day. Based on the estimated mass fraction of perchlorate in Martian regolith, a daily supply of oxygen for one astronaut could be obtained by the complete dissociation of perchlorate contained in 60 kg of regolith. Perchlorate can be extracted from Martian regolith easily with water, which can be recycled for reuse with minimal effort. Because of the large molecular volume and single anionic charge, perchlorate has a low affinity for cations, and, as a result, perchlorate salts are generally highly soluble and completely dissociate into perchlorate and the relevant counter ion in aqueous solutions. Furthermore, perchlorate does not sorb to any significant extent to soils or sediments and, in the absence of any biological interactions, its mobility and fate are largely influenced by the hydrology of the environment [74]. As such, the extraction of perchlorate laden rock simply requires water.

**Figure 4:**
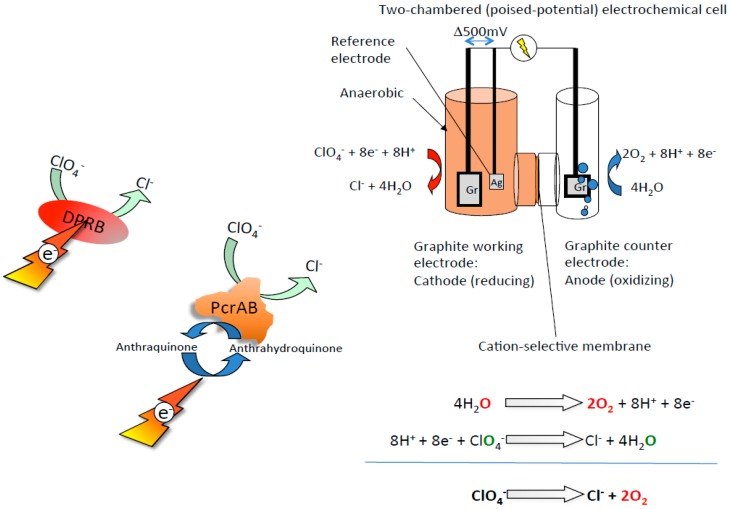
A schematic diagram of a bioelectrical reactor developed for bioelectrochemical oxygen production from perchlorate. Electrochemical consumption of perchlorate in the cathodic chamber mediated by active perchlorate reductase enzymes or lysed dissimilatory perchlorate-reducing bacteria (DPRB) cells are coupled to electrolysis of water releasing pure O_2_ in the anodic chamber.

The method of O_2_ production from the extracted perchlorate takes advantage of electrochemistry combined with enzymatic reduction (Figure 4) previously developed for microbial perchlorate attenuation in a bioelectrical reactor (BER) [56]. Electrochemical consumption of perchlorate in the cathodic chamber mediated by an active perchlorate reductase enzyme can be coupled to electrolysis of water releasing pure O_2_ with no contaminating gases in the anodic chamber. Furthermore, because water is formed in the cathodic chamber at a rate that is identical to that consumed in the anodic chamber (Reactions 1–3) (Figure 4), the system is not limited by water availability.

Reaction 1: 4H_2_O → 2O_2_ + 8H^+^ + 8e^−^

Reaction 2: 8H^+^ + 8e^−^ + ClO_4_^−^ → Cl^−^ + 4H_2_O

Combined Reaction 3: ClO_4_^−^ → Cl^−^ + 2O_2_

The technology is composed of a simple poised potential electrochemical cell containing a cathode and an anode. The redox potential on the cathode can be set at 500–750 mV relative to a reference electrode. The anode voltage is left free floating at ≥1.5 volt, while power can be provided through a photovoltaic cell. The cathode chamber is filled with an aqueous extract of regolith containing perchlorate and amended with pure enzyme or lysed cells with demonstrated perchlorate reduction capacity. The cathode chamber can be further amended with a soluble electron shuttle, such as 2,6-anthraquinone disulphonate (100 μM), to mediate electron transfer from the electrode surface to the active enzyme. Preliminary studies demonstrated the bioelectrochemical reduction of ~1 mM perchlorate over 8 h [56], which equates to the production of ~2 mM O_2_ (or ~45 mL from a 1 L bioreactor), suggesting that some process optimization is required to achieve a goal of 550–650 L of oxygen per day. However, the rate of oxygen production is directly correlated to the rate of perchlorate reduction, which is a function of enzyme concentration in the cathodic chamber, thus it is easy to envision straightforward approaches for rate improvement. The anodic chamber is filled with water and the electrical requirements can either be supplied from photovoltaic cells or from alternative electrical sources. When the water content of the anode chamber is depleted, it can be refilled with water produced in the cathode chamber. While this process may not be suitable to scale up to satisfy the sole needs of a human colony, it could offer an emergency O_2_ alternative, taking advantage of the natural resources of the planet.

## 5. Oil Reservoir Bio-Souring Control

Off-shore petroleum extraction often involves injection of sulfate-rich (~28 mM) seawater into the oil reservoir, which increases the reservoir pressure and facilitates crude oil recovery [75,76]. However, this practice frequently leads to the bio-generation of hydrogen sulfide (H_2_S) by sulfate reducing microorganisms (SRM) [76,77]. This phenomenon, termed bio-souring, poses significant health and operational risks due to the toxic, explosive, and corrosive nature of hydrogen sulfide gas, and has an estimated associated annual cost of $90 billion globally [19,78]. Presently, the most common practice for bio-souring control is the addition of nitrate into the injected seawater to stimulate nitrate respiration [76,77,79,80]. Microbial nitrate reduction is thermodynamically more favorable than microbial sulfate reduction (NO_3_^−^/N_2_ E°′ = +750 mV, SO_4_^−^/HS_2_ E°′ = −217 mV), and, as such, should bio-competitively exclude sulfate reduction [76,79,80]. However, while bio-competitive exclusion may effectively reduce souring in some natural environments, the favorable thermodynamics of nitrate reduction does not exclude the potential for the co-occurrence of sulfate reduction if the electron donor is saturating [81], as is the case in an oilfield. In addition, sulfate reduction can persist in deeper reservoirs as nitrate penetration is limited due to rapid depletion through microbial respiration, as seawater is often a rich source of nitrate reducing organisms [16,76,79,82]. Furthermore, many SRM, including *Desulfovibrio* spp., *Desulfobulbus* spp., and *Desulfomonas* spp., can alternatively use nitrate as a terminal electron acceptor, foreshadowing rapid SRM activity and H_2_S rebound during any interruption of nitrate treatment [83]. 

**Figure 5:**
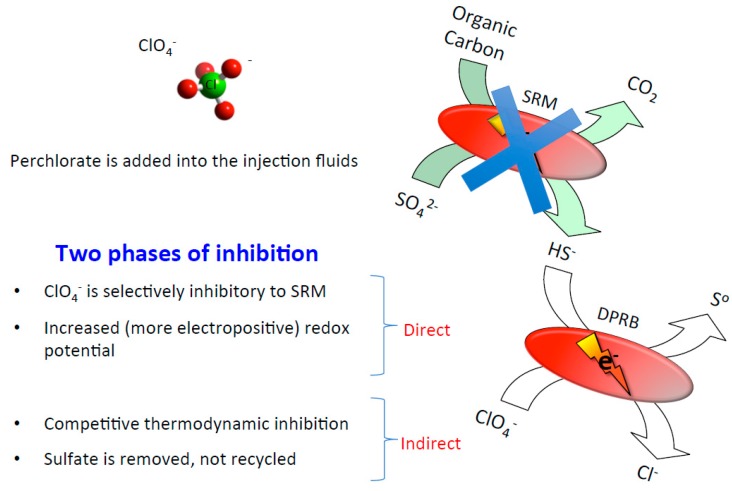
A model illustrating different modes of inhibition of sulfate reducing microorganisms (SRM) by perchlorate and dissimilatory perchlorate-reducing bacteria (DPRB).

Recent studies have exploited perchlorate and DPRB as an alternative method for oil reservoir bio-souring control. Perchlorate treatment has several advantages over nitrate (Figure 5). First, microbial perchlorate respiration is thermodynamically more favorable (E°′ = +797 mV) than either nitrate or sulfate reduction [18,19,75], therefore ensuring the bio-competitive exclusion of SRM by DPRB. Second, sulfide causes electron transport chain short-circuiting in DPRB, in which the perchlorate reductase directly oxidizes sulfide to produce elemental sulfur [84]. Due to the highly conserved nature of the perchlorate reductase, all DPRB tested to date rapidly and preferentially oxidize sulfide, even in the presence of labile physiological electron donors (e.g., acetate, lactate etc.) [75,84]. The DPRB retain the ability to grow on other electron donors once the sulfide is completely biologically removed [75,84]. Finally, perchlorate acts as a competitive inhibitor of the ATP sulfurylase, which is a highly conserved key enzyme in the sulfate reduction pathway [78]. A proof-of-concept study of a flow-through column system mimicking an oil reservoir environment with the potential to sour has confirmed dramatic SRM activity inhibition by the addition of perchlorate [16] at concentrations as low as 3.5 mM [85]. 

## 6. Xenobiotic Bioremediation by DPRB and DCRB

Anthropogenic activities, such as industrial discharges and accidental spills, often result in the release of highly toxic xenobiotics, such as benzene, toluene, ethylbenzene, or xylene (BTEX) into groundwater, soil, or sediments [86,87,88]. Aerobic microorganisms, especially *Pseudomonas* species, utilize oxygen as a co-substrate for oxygenase enzymes to effectively cleave chemically stable aromatic rings [89,90]. However, this combined with the utilization of O_2_ as an electron acceptor for respiration leads to a rapid depletion of oxygen. As such, the contaminated environment becomes anoxic and dominated by anaerobic microbial metabolism [91]. Under anoxic conditions, microbial metabolisms dependent on energy consuming reductive de-aromatization steps to cleave the ring [92,93], and the rate of degradation is significantly slower than when O_2_ is available [94]. 

**Figure 6:**
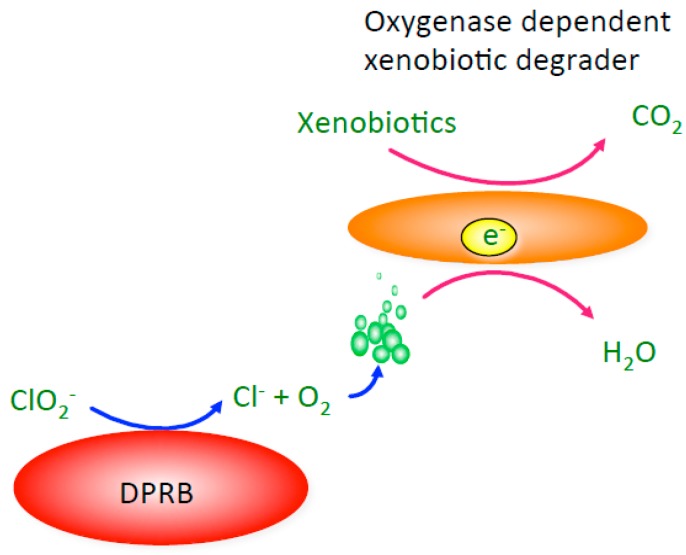
A model of oxygenase-dependent degradation for a xenobiotic degrading co-culture in the absence of O_2_. Pre-requisite oxygen is supplied to the obligately aerobic xenobiotic degrading organism by the dissimilatory (per)chlorate reducing bacterium (DPRB) as a result of the dismutation of added chlorite.

Owing to their unique ability to generate molecular oxygen under anaerobic conditions, it had long been postulated that DPRB and DCRB are capable of stimulating oxygenase-dependent anaerobic metabolisms [19,95,96,97]. This was first shown in anoxic co-cultures of DPRB with obligate aerobic hydrocarbon utilizing *Pseudomonas* spp. [95,96]. In these studies, degradation of benzene and naphthalene was demonstrated under anoxic conditions when the cultures were amended with chlorite. The chlorite was directly dismutated into O_2_ and Cl^−^ by the active DPRB (Figure 6) and the biogenic O_2_ was subsequently available for the aerobic *Pseudomonas* to use as a co-substrate and an electron acceptor for the hydrocarbon metabolism in an oxygenase-dependent manner. An expansion of these studies showed that degradation of both monoaromatic and polycylic aromatic hydrocarbons could be stimulated in contaminated anoxic sediment upon bioaugmentation with DPRB and addition of chlorite [95]. Subsequent studies have similarly demonstrated that methane degradation can be correspondingly stimulated in a co-culture of an aerobic methanotroph with a DPRB [98].

**Figure 7:**
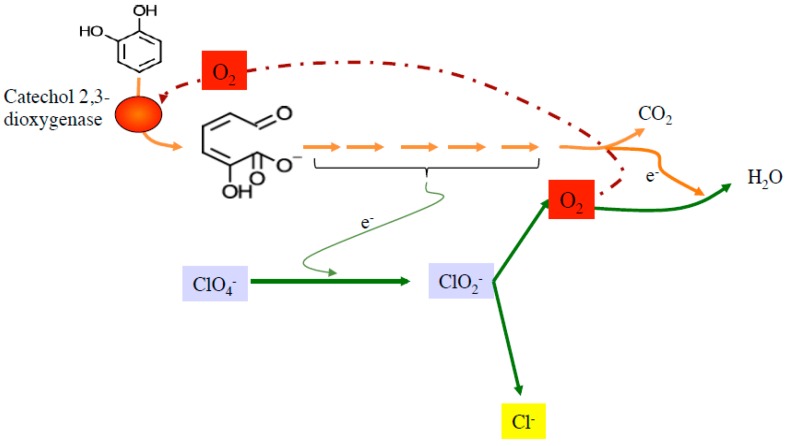
A simplified model illustrating the oxygenase-dependent, anaerobic catechol degradation pathway in the DPRB *Arcobacter* sp. CAB. Green, orange, and red arrows indicates perchlorate reduction pathway, catechol degradation pathway, flow of oxygen, or electrons, respectively.

These findings led to the postulation that DPRB, with the appropriate gene content, may exist that can completely catabolize xenobiotics independently under anoxic conditions in an oxygenase-dependent manner using perchlorate as their sole electron acceptor (Figure 7). Indeed, recent studies have confirmed that the DPRB *Arcobacter* sp. CAB and the DCRB *Dechloromarinus chlorophilus* NSS use aerobic pathways under anaerobic conditions to couple (per)chlorate reduction to the degradation of such aromatic compounds as catechol, phenylacetate, and benzoate [41,42]. Under these conditions, some of the biogenic O_2_ is reused by the cell for the appropriate catabolic pathway (Figure 7). Remarkably, in some of these organisms, internal oxygen consumption is not a prerequisite for aromatic compound degradation coupled to (per)chlorate reduction, as demonstrated by the ability of *Sedimenticola selenatireducens* CUZ to utilize both anaerobic and aerobic catabolic pathways to degrade phenylacetate and benzoate during (per)chlorate respiration [41]. The production of oxygen by DPRB and DCRB makes these organisms excellent candidates for the bioremediation of a broad diversity of recalcitrant xenobiotic compounds under anoxic or oxygen-limiting conditions, both in situ and in bioreactors.

## 7. Genetic Optimization of DPRB and DCRB

The recent development of genetic systems for both DPRB and DCRB allows for the construction of (per)chlorate-reducing organisms with improved phenotypic characteristics [34,35,36,99]. Marker-less gene deletion strains can now be generated by antibiotic selection via suicide plasmid genomic integration, and subsequent plasmid excision via sucrose (*sacB*) or streptomycin (*rpsL*) counter-selection [34,35,36,99]. In addition, gene complementation and enzyme over-expression can be achieved with the pSC101 and pBBR1MCS series of broad range plasmids [34,35,36,99]. Furthermore, bar-coded transposon mutant libraries of the DPRB *Azospira suillum* PS and the DCRB *Pseudomonas stutzeri* PDA are available for rapid gene-fitness profiling under diverse conditions [36,100]. These bar-coded transposon mutant libraries allow for rapid beneficial or detrimental gene identification under different growth conditions (e.g., high salinity, high temperature, high pressure, etc.). Equipped with these molecular tools, engineers have been able to manipulate DPRB and DCRB genomes according to their fitness profiles to achieve desired phenotypes with potential biotechnological application [36,100]. As an example, reactive chlorine species such as chlorite and hypochlorite are produced as toxic intermediates during (per)chlorate respiration. Melnyk and co-workers were able to construct a strain of *A. suillum* that was resistant to reactive chlorine species by knocking out the anti-sigma factor *nrsF*, which is a repressor of the sigma factor *sigF* that activates the expression of hypochlorite scavenging methionine-rich peptide (mrpX) and methionine sulfoxide reductase (yedY1) [100]. The *nrsF* deletion strain showed an increased tolerance and enhanced growth rate with high concentration of chlorate, presumably due to heightened resistance to reactive chlorine species produced as part of the respiratory pathway [100]. 

## 8. Chlorite Mediated Bioreactor Hygiene Control 

The biotechnology industry uses living organisms for the development or biotransformation of its products from raw materials. In 2015, this industry generated $107.7 billion of revenue in the United States (US) alone and has an estimated global market value of $890 billion (www.statista.com/topics/1634/biotechnology-industry). Bioprocessing and biotransformation with bioreactors frequently suffer from contamination with environmental microbes or bacterial phages [101,102,103,104]. Robust long-term operations often rely on costly antibiotic addition, which is economically infeasible for bio-commodity production. A proposed alternative is the use of chlorite and Cld as a biocide/biocide-resistance system for bioprocessing hygiene control. Chlorite is a low-cost biocide with activity against a broad range of microorganisms and viruses [23,24,25,26,27,28,29], and chlorite dismutase provides specific resistance to chlorite. For example, under aerobic conditions, the DPRB *Azospira suillum* PS grew normally in the presence of 40 μM chlorite, while a *cld* gene deletion mutant failed to grow (Coates lab, unpublished observations). Additionally, heterologous expression of Cld in non-(per)chlorate reducing *Shewanella oneidensis* MR-1 protected the strain against chlorite toxicity (Coates lab, unpublished observations). In theory, it should be possible to construct a chlorite hyper-resistant industrial strain by the heterologous expression of Cld. However, one key difficulty is balancing the optimization of Cld activity with chlorite resistance. High Cld activity would lead to rapid chlorite removal, decreasing the selection against contaminant strains. Future studies could utilize directed evolution approaches to optimize and construct a Cld mutant that has low enzymatic activity, but can still confer resistance to the production strain. 

## 9. Chlorite Mediated Aeration Enhancement

The physical properties of molecular oxygen, such as its low aqueous solubility (40 mg/L at 1 atm, 25 °C) and low mass transfer rate through the air-water interface, impose technical challenges in both laboratory enzymology experiments and industry scale bioprocessing [105,106,107,108,109,110]. Owing to the large oxygen demand in high cell density bioreactors, operators sometimes use expensive pure oxygen instead of air for aeration, along with an increased pressure and agitation rate to enhance the oxygen mass transfer [107]. Dassama and co-workers demonstrated that the addition of purified Cld and chlorite in aqueous solution resulted in a “burst” of oxygen generation of >5 mM in less than 1 ms [109]. This efficient enzymatic oxygen-generation system has been applied to increase the yield of unstable (half-life < 1–10 s) oxygenated intermediates [109], and to elucidate the biochemical mechanisms of oxygenases and oxidases [108,111,112]. The addition of purified Cld is implausible in industry-scale bioprocessing plants due to the large volume of industrial bioreactors and the high cost that is associated with Cld purification. However, the aeration problem could be solved by expressing Cld in the process organisms, which, in combination with chlorite addition, results in oxygen generation. Chlorite is extremely cheap (approximately $1 per kg), and only requires mild agitation as a result of its high solubility in water. Preliminary evidence of the potential for this approach was apparent in the demonstration of enhanced oxygenase-dependent benzene and naphthalene degradation under anaerobic conditions by the addition of chlorite and DPRB [95,96]. Further studies are needed for the materialization and validation of this approach.

## 10. Gas Gangrene Treatment

A major medical problem in deep tissue wound therapy relates to the prevention and treatment of gas gangrene. At a basic level, gas gangrene can result from development of anaerobic conditions in deep wounds and infection by obligatory anaerobic *Clostridia* species producing necrotizing tissue toxins. Gas gangrene is generally fatal if left untreated. Furthermore, many *Clostridia* species have developed resistances to a broad range of antibiotics, necessitating the application of alternative treatment technologies. These are often complex, expensive, and of limited success. Apart from amputation of the infected region, current treatments of gas gangrene involve cumbersome hypobaric chambers to increase oxygen levels in infected tissue or “maggot therapy”, in which live maggots are sutured into the wound to graze on the necrotic tissue and *Clostridium* cells. 

**Figure 8:**
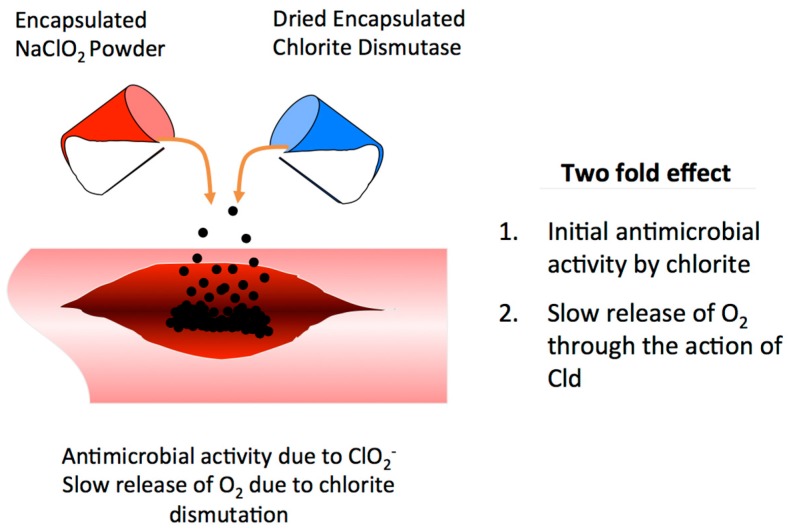
Chlorite dismutase (Cld) and chlorite as a novel alternative treatment for gas gangrene.

A novel alternative treatment was proposed that takes advantage of the activity of Cld purified from DPRB, which produces oxygen in copious quantities from aqueous solutions of chlorite, stored as a dry stable sodium salt under oxic or anoxic conditions. Studies have shown that the purified enzyme is stable and functions optimally at circumneutral pH and 35 °C [113]. From a very simplistic perspective, one can envision the mechanistic basis of such a treatment, whereby the oxygen produced and evolved as gaseous O_2_ [18,96] could inhibit the development and progression of the Clostridial gangrenous infection in deep wounds over the long term (Figure 8). Furthermore, chlorite itself has known biocidal activity against a broad range of microorganisms and viruses [23,24,25,26,27,28,29], and as such would have an immediate antibiotic effect prior to the long term development of oxic conditions. 

## 11. Conclusions

Exciting research in the field of microbial (per)chlorate reduction in the past 20 years has revealed the unique physiological and biochemical characteristics of microbial (per)chlorate reduction and its associated enzymatic and genetic mechanisms. This understanding provides many opportunities to leverage the novel capabilities of these organisms and apply this knowledge to solve outstanding problems in the environmental and biotechnological industries. Aside from perchlorate bioremediation, many of the other topics outlined here require commercial validation, while others are still at the preliminary proof-of-concept stage. Such early stage technologies offer excellent prospects for researchers and industrialists alike, who wish to contribute to the realization and translation of these concepts.
